# Filtering in SPECT Image Reconstruction

**DOI:** 10.1155/2011/693795

**Published:** 2011-06-23

**Authors:** Maria Lyra, Agapi Ploussi

**Affiliations:** Department of Radiology, Radiation Physics Unit, University of Athens, 76, Vas. Sophias Ave., Athens 11528, Greece

## Abstract

Single photon emission computed tomography (SPECT) imaging is widely implemented in nuclear medicine as its clinical role in the diagnosis and management of several diseases is, many times, very helpful (e.g., myocardium perfusion imaging). The quality of SPECT images are degraded by several factors such as noise because of the limited number of counts, attenuation, or scatter of photons. Image filtering is necessary to compensate these effects and, therefore, to improve image quality. The goal of filtering in tomographic images is to suppress statistical noise and simultaneously to preserve spatial resolution and contrast. The aim of this work is to describe the most widely used filters in SPECT applications and how these affect the image quality. The choice of the filter type, the cut-off frequency and the order is a major problem in clinical routine. In many clinical cases, information for specific parameters is not provided, and findings cannot be extrapolated to other similar SPECT imaging applications. A literature review for the determination of the mostly used filters in cardiac, brain, bone, liver, kidneys, and thyroid applications is also presented. As resulting from the overview, no filter is perfect, and the selection of the proper filters, most of the times, is done empirically. The standardization of image-processing results may limit the filter types for each SPECT examination to certain few filters and some of their parameters. Standardization, also, helps in reducing image processing time, as the filters and their parameters must be standardised before being put to clinical use. Commercial reconstruction software selections lead to comparable results interdepartmentally. The manufacturers normally supply default filters/parameters, but these may not be relevant in various clinical situations. After proper standardisation, it is possible to use many suitable filters or one optimal filter.

## 1. Introduction

Tomography is a noninvasive imaging technique that is used to generate cross-sectionals images of a three dimensional (3D) object without superimposing tissues. Tomography can be categorized in transmission tomography such as computed tomography (CT) and emission tomography like single photon emission computed tomography (SPECT) and positron emission tomography (PET). Computed tomography is a technique based on X-ray transmission through a patient to create images of sections (slices) of the body. Photon emission computed tomography and positron emission tomography provide 3D image information about the radionuclide injected into the patient that shows the metabolic and physiological activities within an organ. 

In tomographic techniques, projections are acquired from many different angles around the body by one or more rotating detectors. These data are then reconstructed and put together to form 3D images of the body. The reconstruction of tomographic images is made by two methods: filtered backprojection and iterative methods [1].

The quality of the final tomographic image is limited by several factors. Some of these are the attenuation and scatter of gamma ray photons, the detection efficiency and the spatial resolution of the collimator-detector system [2]. These factors have as a result poor spatial resolution, low contrast, and high noise levels. Image filtering techniques are very important in tomography as they strongly affect the quality of the image.

Image filtering is the term used for any operation that is applied to pixels in an image. It is a mathematical process by which images are suppressed in noise and also includes smoothing, edge enhancement and resolution recovery.

 Filters are used during reconstruction and applied to data in frequency domain. The goal of the filtering is to compensate for loss of detail in an image while reducing noise. The application of filters is the most common method to reduce high-frequency noise component in projection images. In this way, filters can greatly improve the image resolution and limit the degradation of the image. There are several types of filters used in medical imaging and the choice of the appropriate filter is a headache in clinical practice [3]. 

The aim of this article is to describe the most commonly used filters in SPECT imaging by analytical techniques. These filters are applied in filtered back projection (FBP) reconstruction techniques. Filtering can also be considered as a postprocessing step in iterative reconstruction. Though many times iteratively reconstructed images need to be postfiltered, as they tend to be noisy, special dedicated iterative filters are not established yet to be included in commercial software. We present also choices of filters for some SPECT examinations that are common in clinical routine, as they are suggested in the literature. 

## 2. SPECT Image Acquisition

Nowadays single-photon emission computed Tomography (SPECT) is widely used in nuclear medical imaging. SPECT is a nuclear medical tomographic imaging technique that represents the distribution of an administered radioactive tracer within an organ. The radiopharmaceutical emits single gamma ray photons. SPECT systems use one or more gamma camera mounted on a gantry so that the detector can rotate around the patient. From the acquired one dimensional projection data from different views around the object, two dimensional (2D) planar projections images are obtained in many evenly spaced angles around the patient and provide an estimate of 3D distribution of the radiotracer using image reconstruction from multiple projections. Some systems acquire the images during their rotational movement, while others stop and record (stop and shoot) an image at selected angles. In SPECT the projections images are generally acquired over a full 360-degree or 180-degree arc (in case of SPECT myocardium perfusion study or kidneys SPECT acquisition), on a matrix of 64∗64 or 128∗128 pixels. Typically the projections are acquired every 3–6 degrees and the total scan time is about 15–20 minutes. The 2D projection-images are first corrected for nonuniformities and then mathematical algorithms are used to reconstruct 3D matrices of selected planes from the 2D projection data. 

## 3. SPECT Image Reconstruction

The purpose of reconstruction algorithms is to calculate an accurate 3D radioactivity distribution from the acquired projections. There are two methods to reconstruct SPECT images, either iteratively or by FBP technique. 

### 3.1. Iterative Reconstruction Method

Iterative reconstruction starts with an initial estimate of the image [4]. Most of the times the initial estimate is very simple, for example a uniform activity distribution. Then a set of projection data is estimated from the initial estimate using a mathematical process called forward projection. The resulting projections are compared with the recorded projections and the differences between the two are used to update the estimated image. The iterative process is repeated until the differences between the calculated and measured data are smaller than a specified preselected value. The iterative reconstruction methods include algebraic methods like the algebraic reconstruction technique (ART) and statistical algorithms like maximum likelihood expectation maximization (MLEM) or ordered-subsets expectation maximization (OSEM) [1]. 

### 3.2. Filtered Backprojection Method (FBP)

FBP is an analytical method that is still the most widely used in clinical SPECT because of its simplicity, speed, and computational efficiency. It consists of two steps: filtering of data and back projection of the filtered data [5].

In 2D acquisition, each row of projections represents the sum of all counts along a straight line through the depth of the object being imaged. Back projection technique redistributes the number of counts at each particular point back along a line from which they were originally detected. This process is repeated for all pixels and all angles. The limited number of projection sets has as a result the creation of a star artifact and the blurring of the image. To eliminate this problem the projections are filtered before being back projected onto the image matrix. It has to be noticed that the backprojection process is taken place in spatial domain while data filtration is done in the frequency domain. 

### 3.3. Image SPECT Filtering

The image restoration process is an example of Fourier spectrum filtering. Once a Fourier Spectrum has been generated for an image, it can be filtered so that certain spatial frequencies can be modified, enhanced or suppressed. This filtered spectrum can then be inverse transformed to generate a filtered image with, for example, sharpened or smoothed features. A feature we need to consider in more detail is the spatial frequency nature of the image data itself. Images are generally sampled digitally using a square matrix composed of pixels, the size of which dictates how well a digital image approximates its analogue counterpart.

The filters used in FBP are simply mathematical equations that vary with frequency. The filters used in SPECT imaging can vary to achieve different purposes such as star artifact reduction, noise suppression, or signal enhancement and restoration.

The choice of filter for a given image reconstruction task is generally a compromise between the extent of noise reduction and fine detail suppression (and of contrast enhancement in some cases) as well as the spatial frequency pattern of the image data of interest. 

#### 3.3.1. Filtering to Reduce the Star Artifact

**Ramp Filter**

The ramp filter is a high pass filter that does not permit low frequencies that cause blurring to appear in the image. In frequency domain, its mathematical function is given by (1).

(1) $$H_{R}\left(k_{x},k_{y}\right)=k={\left(k_{x}^{2}+k_{y}^{2}\right)}^{1/2},$$

where *k*_*x*_, *k*_*y*_ are the spatial frequencies.

**Figure 1:**
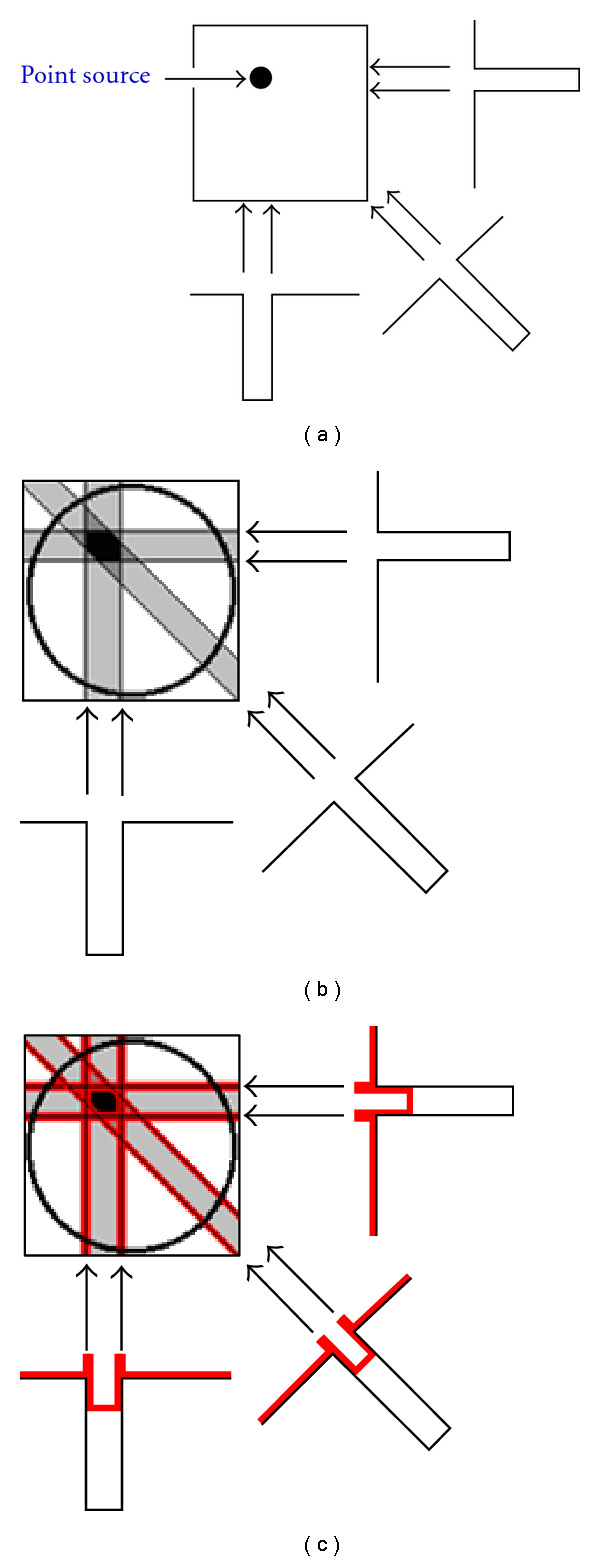
A simple representation of filtered back projection. (a) Acquisition of three projections. (b) Backprojected projections. (c) Filtered backprojected projections.

The Ramp is a compensatory filter as it eliminates the star artifact resulting from simple backprojection. Because the blurring is only appeared in the transaxial plane, the filter, is only applied in that plane [5]. The filter as shown in Figure 1(a)), is linearly proportional to the spatial frequency. High pass filters sharpen the edges of the image (areas in an image where the signal changes rapidly) and enhance object edge information. A severe disadvantage of high pass filtering is the amplification of statistical noise present in the measured counts. In order to reduce the amplification of high-frequencies the ramp filter is always combined with a low-pass filter.

#### 3.3.2. Filtering to Reduce Noise

The common method to reduce or remove statistical noise in a SPECT image is the application of smoothing filters. These filters are low-pass filters which allow the low frequencies to be retained unaltered and block the high frequencies. Low-pass filters are characterized mainly by two parameters—the “cut-off frequency” and the “Order” (or the “Power”). The cut-off frequency (or roll-off frequency) defines the frequency above which the noise is eliminated. The filter function is defined to be zero for all frequencies above cut-off frequency. The Nyquist (Nq) frequency—the highest frequency that can be displayed in an image—is apparently the highest cut-off frequency for a filter. The cut-off frequency is expressed in cycles per pixel or as a fraction of the Nq frequency. The cut-off frequency varies typically from 0.2 to 1.0 times the Nq frequency. The value of the cut-off frequency determines how the filter will affect both image noise and resolution. A high cut-off frequency will improve the spatial resolution and therefore much detail can be seen but the image will remain noisy. A low cut-off frequency will increase smoothing but will degrade image contrast in the final reconstruction. 

The parameter Order controls the slope of the filter function and characterizes the steepness of the roll off. A high order will result in a sharp fall. Sometimes, the term power instead of order is used. The power is twice the order. 

There is a number of low-pass filters that are available for SPECT reconstruction. The most commonly used are discussed below.

**Butterworth Filter**

**Figure 3:**
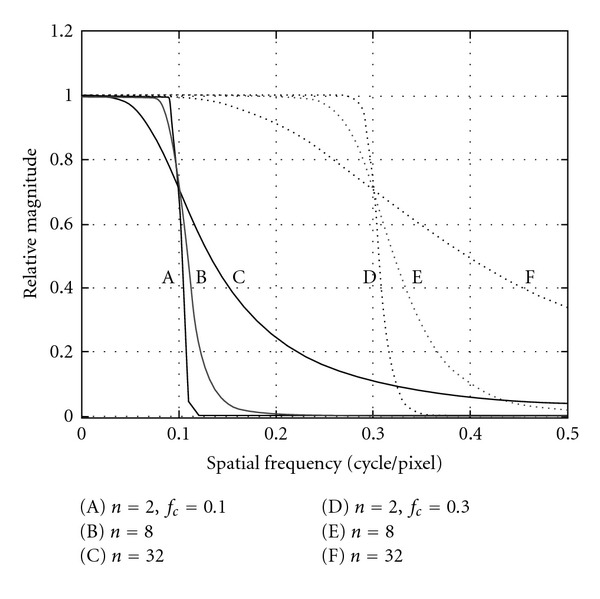
Butterworth smoothing filter six curves by different *f*_*c*_ and *n* parameters (equation (2)). A, B, C curves created by critical frequency *f*_*c*_ = 0.1 c/pixel and order *n* equal to 2, 8, 32 correspondingly. D, E, F curves created by critical frequency *f*_*c*_ = 0.3 c/pixel and order *n* equal to 2, 8, 32 similarly [6].

Butterworth filter is the more usual choice in nuclear medicine. The butterworth filter is a low-pass filter. It is characterized by two parameters: the critical frequency which is the point at which the filter starts its roll off to zero and the order or power [6]. As it is mentioned earlier, the order changes the slope of the filter. Because of this ability of changing not only the critical frequency but also the steepness of the roll-off, the butterworth filter can do both, smoothes noise and preserves the image resolution. A butterworth filter in spatial domain is described by:

(2) $$B\left(f\right)=\frac{1}{\left[1+{\left(f/f_{c}\right)}^{2n}\right]},$$

where *f* is the spatial frequency domain, *f*_*c*_ the critical frequency and *n* the Order of the filter (Figure 3).

**Figure 4:**
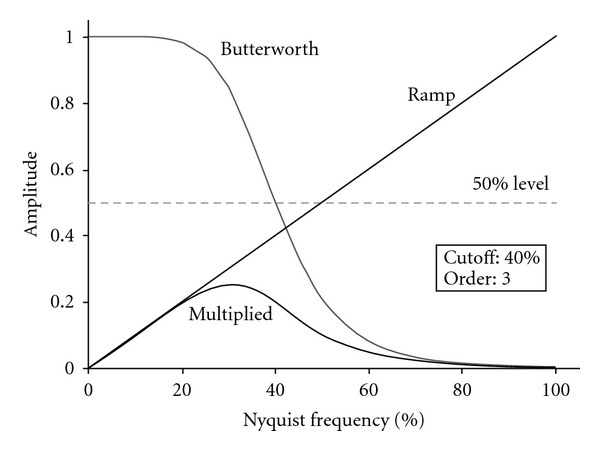
Illustration of the Butterworth filtering process. A Ramp function and a Butterworth function (of Order 3 and cut-off frequency 40% of Nq frequency) are multiplied to form the Fourier filter used in the FBP process. Generated by Kieran Maher, 2006, accessed in http://en.wikibooks.org/wiki/File:NM16_14.gif.

A ramp function and a butterworth function of variable order and cut-off (critical) frequency, are multiplied to form the fourier filter used in the FBP process (Figure 4).

**Hanning Filter**

**Figure 5:**
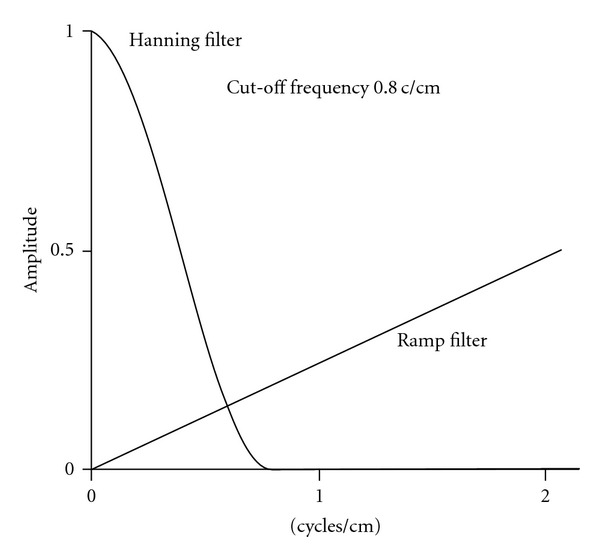
Hanning filter and Ramp in FBP reconstruction.

The Hanning filter is a relatively simple low-pass filter which is described by one parameter, the cut-off (critical) frequency (Figure 5) [7].

The Hanning filter is defined in the frequency domain as follows:

(3) $$H\left(f\right)=\{\begin{array}{cc} 0.50+0.50\;\cos\;\;\left(\frac{\pi f}{f_{m}}\right), & 0\leq \left|f\right|\leq f_{m},\\ 0, & \text{otherwise}, \end{array}$$

where *f* are the spatial frequencies of the image and *f*_*m*_ the cut-off (critical) frequency.

In signal processing, the Hann window is a window function, called the Hann function, named after Julius Ferdinand von Hann, an Austrian meteorologist. The use of the Hann window is called “Hanning”, as a signal to apply the Hann window to a digital signal processing. http://en.wikipedia.org/wiki/Hann_function.

The Hanning (Hann) filter is very effective in reducing image noise as it reaches zero very quickly; however, it does not preserve edges (Figure 5).

**Hamming Filter**

The Hamming filter is also a low pass filter, which presents a high degree of smoothing, named after Richard Wesley Hamming, an American mathematician famous in computer science. As the Hanning filter, it has only a single parameter to describe its shape, the cut-off frequency. The mathematical definition is shown as (4) [7].

(4) $$H\left(f\right)=\{\begin{array}{cc} 0.54+0.46\;\cos\;\;\left(\frac{\pi f}{f_{m}}\right), & 0\leq \left|f\right|\leq f_{m},\\ 0, & \text{otherwise}, \end{array}$$

where **f** are the spatial frequencies of the image and *f*_*m*_ the cut-off frequency.

As it can be observed the only difference with the Hanning filter is on the amplitude at the cut-off frequency.

**Parzen Filter**

The Parzen filter is another example of low pass filter and it is defined in the frequency domain as [7],

(5) $$\begin{array}{c} \left|f\right|-6\left|f\right|{\left(\frac{\left|f\right|}{f_{m}}\right)}^{2}\times \left(1-\frac{\left|f\right|}{f_{m}}\right)\;\left(\left|f\right|<\frac{f_{m}}{2}\right),\\ P\left(f\right)=\{\begin{array}{cc} 2\left|f\right|{\left(1-\frac{\left|f\right|}{f_{m}}\right)}^{3}, & \left(\frac{f_{m}}{2}<\left|f\right|<f_{m}\right),\\ 0, & \left(\left|f\right|\geq f_{m}\right), \end{array} \end{array}$$

where *f* are the spatial frequencies of the image and *f*_*m*_ the cut-off frequency.The Parzen filter is the most smoothing filter; it eliminates high-frequency noise, but it also degrades the image resolution [3].

**Shepp-Logan Filter**

The Shepp-Logan is one more filter that belongs to the family of low pass filters. Its mathematical equation is shown as (6) [8].

(6) $$S\left(f\right)=\frac{2f_{m}}{\left[\pi \left(\sin\left|f\right|\pi /2f_{m}\right)\right]}.$$

The Shepp-Logan filter produces the least smoothing and has the highest resolution.

Numerous types of filters exist, and all filters aim, except for the restoration filters, at reducing frequency information through an amplitude-adjusting function between 0 and 1 Nq. The interpretation and comparison of SPECT studies is beclouded by the use of too many different filter types.

**Figure 6:**
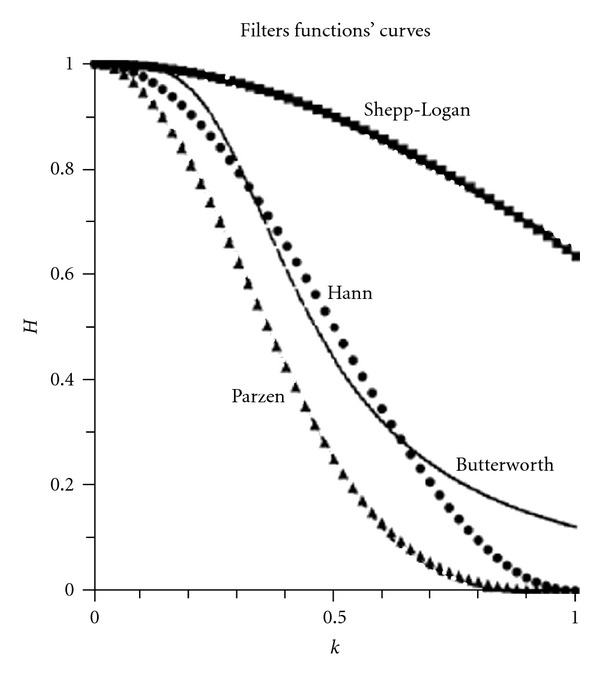
Shepp-Logan, Butterworth, Hann, Parzen filter functions'. (from Van Laere et al., (2001), modified ) [3].

Optimal parameters have been calculated [3] for Butterworth or Hanning filters to match the shape of various existing filter types. Butterworth filters cannot approximate any other kind of filter shape since the amplification of the high-frequency components always asymptotically approaches zero, whereas for the Hann filter, high-frequency components can be set to zero. This is demonstrated for the approximation of a Hann filter by Butterworth matching (Figure 6). A Shepp-Logan filter can be very accurately matched to a Butterworth filter with the appropriate parameters. A Parzen filter is closely matched by a Hann filter with cut-off 1 (Figure 6).

From the practical point of view, all filter shapes can be fairly accurately addressed by a specific cut-off/order/restoration combination of Butterworth and Hann filtering.

#### 3.3.3. Filtering to Enhance the Signal

A low-pass filter may smooth image to a high degree that does not permit discerning small lesions, leading to contrast loss. For this reason a third class of filters, called enhancement or restoration filters, is used in SPECT imaging. The restoration filters enhance the signal with a simultaneous reduction of noise without resolution lost. Metz and Wiener are two types of resolution recovery filters that have been used in nuclear medicine image processing.

**Metz Filter**

Metz filter is a function of modulation transfer function (MTF), and it is based on the measured MTF of the gamma camera system. The MTF describes how the system handles or degrades the frequencies. The Metz restoration filter is defined in the frequency domain as

(7) $$M\left(f\right)=\text{MTF}{\left(f\right)}^{-1}\left[l-{\left(l-\text{MTF}{\left(f\right)}^{2}\right)}^{x}\right],$$

where *f* is the spatial domain and *x* is a parameter that controls the extent to which the inverse filter is followed before the low-pass filter rollsoff to zero [9]. Equation (7) is the product of the inverse filter (first term) and a low pass filter (second term).

**Figure 7:**
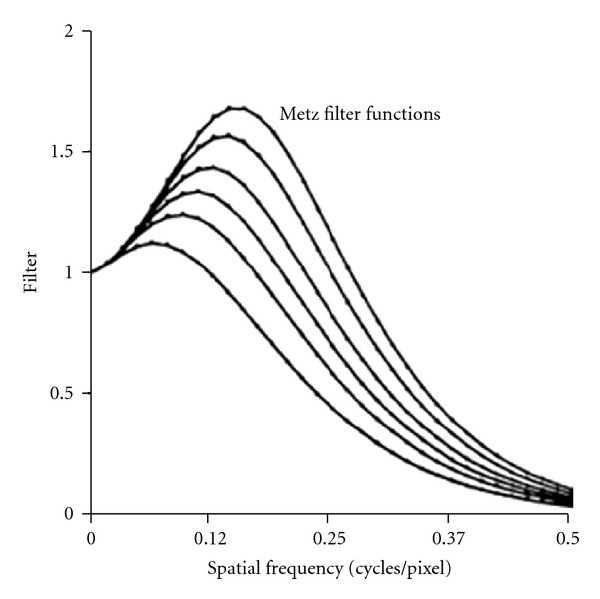
Plot of Metz filter for total counts of 20.000, 50.000, 100.000, 200.000, 500.000, and 1 million counts from lowest to highest curve [10].

The Metz filter is count dependent.  Figure 7 shows the Metz filter plotted for six different total image counts [10].

From Figure 7 results that, as the counts increase, more resolution recovery occurs (filter rises farther above 1.0), together with less suppression (filter moves farther to right) [10].

**Wiener**

The Wiener filter is based on the signal-to-noise ratio (SNR) of the specific image. The one dimensional frequency domain form of the Wiener filter is defined as

(8) $$W\left(f\right)={\text{MTF}}^{-1}\times \frac{{\text{MTF}}^{2}}{\left({\text{MTF}}^{2}+N/O\right)},$$

where MTF is the modulation transfer function of the imaging system, *N* is the noise power spectrum, and O is the object power spectrum [11]. As with the Metz filter, the Wiener is the product of the inverse filter (which shows the resolution recovery) and the low pass filter (which shows the noise suppression). In order to apply the Wiener filter it is necessary to know a priori the MTF, the power spectrum of the object and the power spectrum of the noise. It has to be noted that is, impossible to know exactly the MTF or the SNR in any image. As a result, the mathematical models used to optimize both Metz and Wiener filters are uncertain [3].

### 3.4. Parameters Determining the Choice of the SPECT Filter Type

Today, gamma camera systems offer a choice of various filters which may be selected depending on the type of examination. The filter choice depends on [3, 12]:

- the energy of the isotope, the number of counts and the activity administration.
- the statistical noise and the background noise level.
- the type of the organ being imaged.
- the kind of information we want to obtain from the images.
- the collimator that is used.

The choice of the filter must ensure the best compromise between the noise reduction and the resolution in the image. 

## 4. Type of Filters Depending on Type of Study

**Figure 8:**
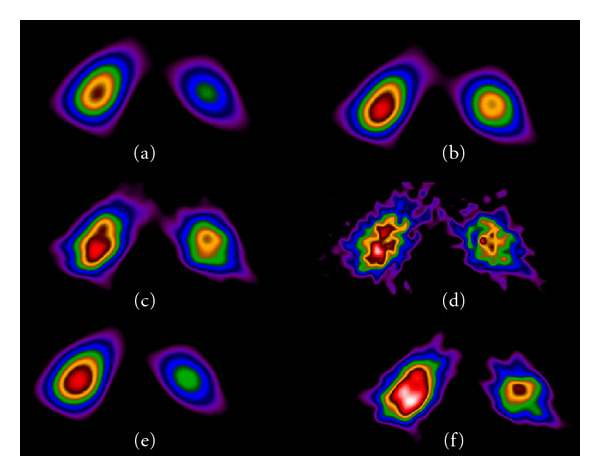
Transverse slices of kidneys'. Various pre- postfiltered in FBP reconstruction were applied with different effects on the images Filters used were (a) prefilter Hanning (cut-off 0.8 cm^−1^), postfilter Ramp. (b) prefilter Butterworth (cut-off 0.5 cm^−1^ power value 10), postfilter Ramp. (c) prefilter Butterworth (cut-off 0.8 cm^−1^, power value 10), postfilter Ramp. (d) only Ramp prefilter applied—no other smoothing filter. (e) prefilter Ramp, postfilter Hanning (cut off 0.8 cm^−1^) (f) prefilter Ramp, postfilter Butterworth (cut-off 0.8 cm^−1^, power value 10). *Study has been completed in Radiation Physics Unit, Department of Radiology, University of Athens*.

The selection of the proper filter and the determination of filter parameters is a major problem in clinical routine. In this section, the filters used for widespread applications of SPECT are listed as they are described in the literature. Image filtering is an important, though mostly subjectively applied, image-processing parameter. It is shown that ramp, Hann and Butterworth filters are the most commonly used image pre- and postprocessing filters. In many clinical evaluations, literature does not provide useful information for specific parameters of the imaging filters. In most clinical routine cases the choice of a filter is done empirically, and the use of limited filter types, in an attempt to standardise image-processing approaches, may lead to better diagnostic compatibility and interpretation of interdepartmental results. In Figure 8, the effect of pre- or postfiltering by ramp-Hanning-Butterworth filters is shown, in coronal slices of a SPECT renal study of a 6-month old boy. 

### 4.1. Cardiac SPECT

Cardiac SPECT has an important clinical role in the detection of myocardial perfusion and the diagnosis of ischemic heart disease. The commonly used radiotracers for cardiac SPECT are Thallium-201 (^201^Tl) and Technetium-99m (^99m^Tc) labeled agents such as ^99m^Tc-Sestamibi and ^99m^-Tetrafosmin. In clinical practice, Hanning filters were preferred for ^201^Tl images and Butterworth for ^99m^Tc images [2]. In the literature, there are extensive studies about the determination of the appropriate filter for myocardial SPECT imaging.

Takavar et al. (2004) [13] studied the determination of the optimum filter in ^99m^Tc myocardial SPECT using a phantom that simulates the heart's left ventricle. Filters such as Parzen, Hanning, Hamming, and Butterworth and a combination of their characteristic parameters were applied on the phantom images. The cut-off frequency of 0.325 Nq and 0.5 Nq gave the best overall result for Hanning and Hamming filters, respectively. For Butterworth filter order 11 and cutoff 0.45 Nq gave the best image quality and size accuracy.

A determination of the appropriate filter for myocardial SPECT was conducted by Salihin Yussoff and Zakaria [7]. The filters' functions evaluated in this study included Butterworth, Hamming, Hanning, and Parzen filters. From these filters, 272 combinations of filter parameters were selected and applied to the projection data. The study suggested that Butterworth filter succeeds the best compromise between SNR and detail in the image while Parzen filter produced the best accurate size. 

The same group [14] investigated the relationship between the optimum cut-off frequency for Butterworth filter and lung-heart ratio in ^99m^Tc myocardial SPECT. A linear relationship between cut-off frequency and lung-heart ratio had been found which shows that the lung-heart ratio must refer in each patient in order to choose the optimum cut off frequency for Butterworth filter.

Links et al. (1990) [11] examined the affect of Wiener filter in myocardial perfusion with ^201^Tl SPECT. The study wad done in 19 dogs and showed that Wiener filter improves the quantization of regional myocardial perfusion deficits.

In a ^201^Tl gated SPECT study in patients with major myocardial infraction [15], a Butterworth filter of order 5 with six cut-off frequencies (0.13, 0.15, 0.20, 0.25, 0.30, 0.35 cycle/pixel) were successively tested. The report showed that filtering affect end-diastolic volume (EDV), end-systolic volume (ESV) and left ventricular ejection fraction (LVEF). Marie et al. (2005) [16] suggested that the best results for cardiac gated SPECT image reconstruction with ^201^Tl were achieved using a Butterworth filter with an order of 5 and cut-off frequency 0.30 cycles/pixel.

### 4.2. Brain SPECT

Brain SPECT is a powerful diagnostic tool for evaluating neurologic and psychiatric diseases. Brain SPECT provides a measure of cerebral blood flow (CBF) and it is very useful for functional imaging of subcortical structures of the brain. There are currently two commercial radiotracers for brain SPECT imaging: Iodium-123 labeled amphetamine (IMB) and ^99m^Tc hexamethylpropyleneamine oxime (HMPAO). Due to the low SNR in this type of study the choice of the optimum filter is difficult enough.

Groch and Erwin (2000) [5] showed that the most suitable filter for ^99m^Tc-HMPAO brain SPECT study is the Butterworth filter with order 10 and 0.5 Nq cut-off frequency. This filter gave the best compromise between noise and spatial resolution with respect to Hamming filter. 

In another report [17], the optimization of Butterworth filter for brain SPECT imaging was studied. The aim of the work was to find a relationship between the total counts and the optimal cut off frequencies of the Butterworth filter. The study proved that as the number of total counts increased the optimal cut-off frequency linearly increased within a specific range of counts.

Raeisi et al. (2007) [18] examined Ramp, Shepp-Logan, Hanning, Hamming, Butterworth, Metz, and Wiener filters in data from brain SPECT. The study suggested that both Metz and Wiener give the maximum resolution and contrast while Butterworth generate the best image quality.

### 4.3. Other SPECT Studies

Although myocardial and brain SPECT studies are the most widespread applications in tomographic nuclear medicine examinations, there are several other organs' SPECT studies that were not very commonly used in clinical routine. In this time, SPECT diagnostic and quantitative value is recognised as complementary assistance in the clinical diagnostic procedures, and accurate volume estimations by SPECT are feasible when accurate corrections are performed [19]. Some of them are bone, liver, lungs, kidneys, and thyroid SPECT examinations. For these applications, the most popular filters are Butterworth and Hanning with different critical frequency values for Hanning and various power and critical frequencies with Butterworth filter. In many clinical cases, information for specific parameters is not provided and filters' parameters findings cannot be evaluated and categorized per organ study.

**Bone**

SPECT is an important diagnostic tool in nuclear medicine for evaluating a detail image of the bones and especially for detecting malignant. There are limited reports in the literature for the appropriate filter in bone SPECT. However, Butterworth filter seems to provide more efficiently anatomic details than other types of filters [20, 21]. Image-dependent Metz filters have been shown to provide consistently good image quality for bone study [22].

**Liver**

disease can be imaged using SPECT to determine the existence of sarcoma, hepatic tumour, haemangiomas, metastases, cyst, glycogen storage disease, and so forth, using ^99m^Tc sulfur colloid (SC) [23]. In a SPECT study for the anatomy of normal liver, Carrasquillo et al. (1983) [24] suggested a modified Butterworth-ramp filter for the image reconstruction. King et al. (1984) [10] showed that two dimensional filtering, before and after reconstruction, using the Metz and Wiener filters can improve significantly the quality of liver SPECT images. Because of the high-count rate and the high SNR in liver SPECT images, filters with a high cut-off frequency are recommended to be used [3].

**Figure 9:**
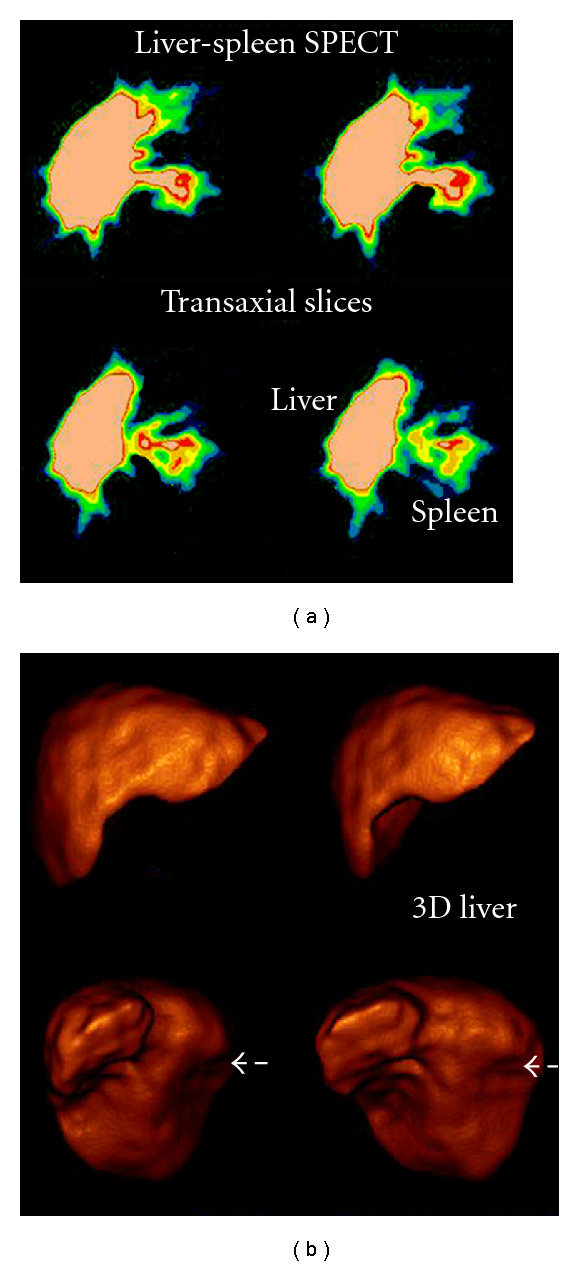
A liver-spleen study SPECT and 3D SPECT. (a) Transverse slices, prefiltered only by Ramp filter could emerge a small piece of the ruptured spleen. (b) 3D SPECT reconstruction by ramp-Hanning filters (Hanning cut-off frequency equal to 0.8 cycles/cm), a threshold 25% and 15% gradient, could show the liver and a short fracture in the middle of right lobe of the liver but misses any residual spleen fragment. (*Study has been offered by Radiation Physics Unit, Department of Radiology, University of Athens)*.

Figure 9 shows a young boy's liver-spleen study following a car accident, searching for residual spleen pieces.

**Figure 10:**
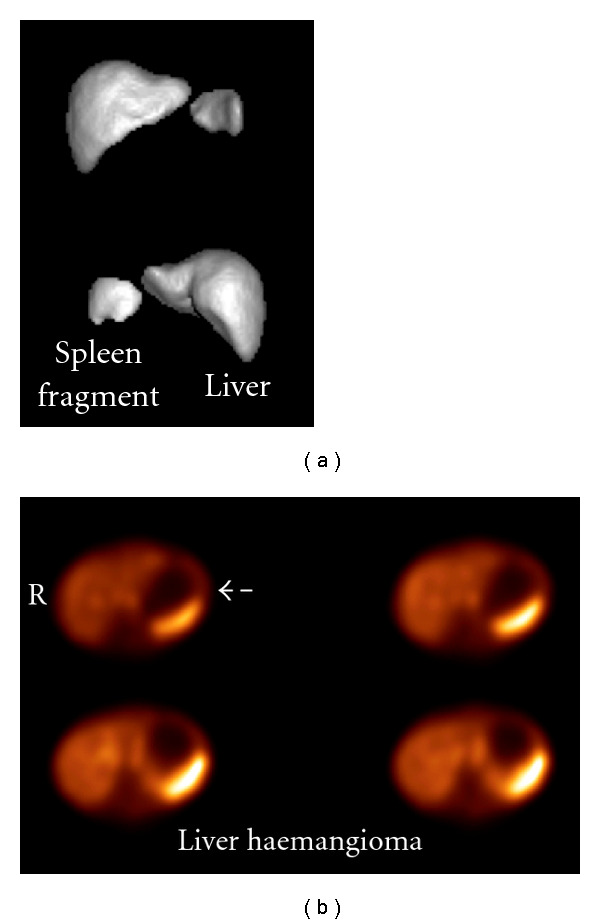
3D liver surface images. (a) Liver and spleen fragment caused by accident. Images reconstructed by FBP, prefiltered by Hanning (critical value 0.8 cm^−1^) and ramp filter. (b) Haemangioma of liver. Images reconstructed by FBP, prefiltered by Butterworth (cut off 0.5 cm^−1^, power factor = 10) and ramp filter. (*Studies offered by Radiation Physics Unit, Department of Radiology, University of Athens)*.

Two more cases of liver 3D SPECT images, reconstructed by FBP, and different filters applied in (Figure 10).

**Renal**

SPECT by ^99m^Tc-DMSA is recommended to be used instead of or complementarily to planar scintigraphy as the preferable study to help especially in paediatrics with early diagnosis, followup, and monitoring of the effects of treatment in acute pyelonephritis and possible scars formation [25–28].

In a renal SPECT study with ^99m^Tc-DMSA, De Sadeleer et al. (1996) [25] suggested the use of a Butterworth filter with an order of 7 and a cut-off frequency of 0.55 Nq, for the reconstruction of the projection data. According to Groshar et al. (1997) [26], a Hanning filter with a cut-off frequency of 0.5 cycle/cm was applied in the data, in a kidney SPECT imaging with ^99m^Tc-DMSA for best results.

In a study, by Yen et al. (1996) [27] for monitoring paediatric acute pyelonephritis by ^99m^Tc-DMSA renal SPECT imaging, a Metz prefilter was applied and transverse images were reconstructed with back projection and a ramp filter to show signs of acute pyelonephritis not indicated in planar renal images.

A semiquantitative evaluation of cortical damage to the kidneys, in children, was performed by tomographic renal Tc99m-DMSA studies. Reconstruction by FBP used Hanning filter (critical frequency 0.8 cm^−1^ and attenuation correction 0.12 cm^−1^). The result of this procedure was the calculation of three integrated over volume (IOV) indices that offer a quantitative comparison of the planar, tomographic, and 3D reconstructed images [28].

Recently, in a ^99m^Tc-DMSA renal cortical SPECT imaging study by dual head gamma camera, reconstruction was performed similarly on both cameras using a Hann prefilter (cutoff frequency, 0.9 cm^−1^; order, 0) and a Butterworth postfilter (cutoff frequency, 0.5 cm^−1^; order, 10) with two iterations and 10 subsets for the detection of renal parenchyma focal defects [29].

Sheehy et al. (2009) have compared two methods of reconstructing ^99m^Tc-dimercaptosuccinic acid (DMSA) renal SPECT data—ordered subset expectation maximization with OSEM-3D and FBP—in children in terms of improving image quality and reducing the radiopharmaceutical activity and radiation dose. Authors do not indicate the filters and relative parameters that were applied during FBP [30]. OSEM-3D was described by Römer et al. (2006) as an iterative SPECT reconstruction algorithm that is performed by using OSEM with 3-dimensional resolution recovery, which is applied in the *x*, *y*, and *z* directions. They had found that this approach, as compared with FBP, substantially improves SPECT image quality and can be performed with fewer gamma photon counts [31].

**Lungs**

SPECT techniques were, up to few years ago, used in clinical diagnosis only by a limited number of centers. Given the improvements in sensitivity and diagnostic accuracy that has generally accompanied the transition from two-dimensional planar to three-dimensional (3D) imaging, SPECT technique in ventilation/perfusion (V/P) scintigraphy historically, one of the most commonly performed diagnostic studies in nuclear medicine, is superior in contrast resolution and improved anatomical detail compared with V/P perfusion scintigraphy, in the diagnosis of perfusion embolism [32].

Gutte et al. (2010) concluded that V/Q SPECT should be preferred in diagnosing of perfusion embolism. In their study, SPECT datasets were attenuation corrected using the low-dose CT acquisition with iterative reconstruction using the software Autospect+ and Astonish with three iterations and 16 subsets [33].

An automated linear registration algorithm based on the maximization of mutual information was applied by Reinartz et al. (2006) to the V/Q scans using the Hermes Multimodality software including processing filters. This automated analysis leads to a significant improvement in the detection rate of pathologic lesions [34].

Harris et al. (2007) show that objective analysis of SPECT V/Q scintigraphy provides a useful tool to help reduce the number of nondiagnostic scintigraphy results. In their study SPECT data were reconstructed to 128 slices using Ordered Subset Expectation Maximization iterative reconstruction (8 iterations, 4 subsets), and smoothed with a postreconstruction three-dimensional Butterworth filter [35].

A comparison of usefulness of SPECT versus planar lung scintigraphy in suspected pulmonary embolism, in daily practice was completed by Weinmann et al. (2008). Reconstruction of coronal, sagittal and transverse slices was done by FBP followed by two iterations with a 5-order Butterworth filter and a cut-off frequency at 0.45 Nq [36].

**Figure 11:**
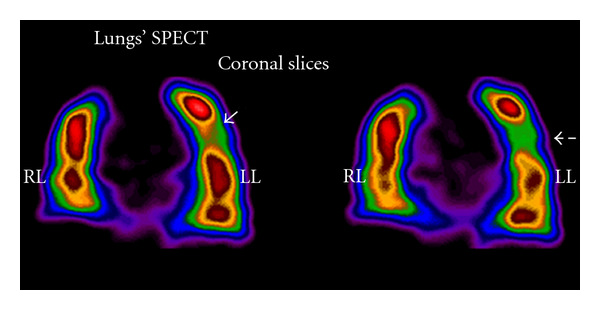
Two sequential coronal slices indicating mild pulmonary embolism in left lung lobe (LL) not indicating in planar images. Image reconstruction has been completed by FBP and Butterworth filter (critical frequency 0.5, order 10). *Courtesy of M. Gavrilelli (MSc, “Medical Imaging Center” Athens, Greece)*.

An example of lungs' perfusion embolism study by SPECT is following (Figure 11).

**Figure 12:**
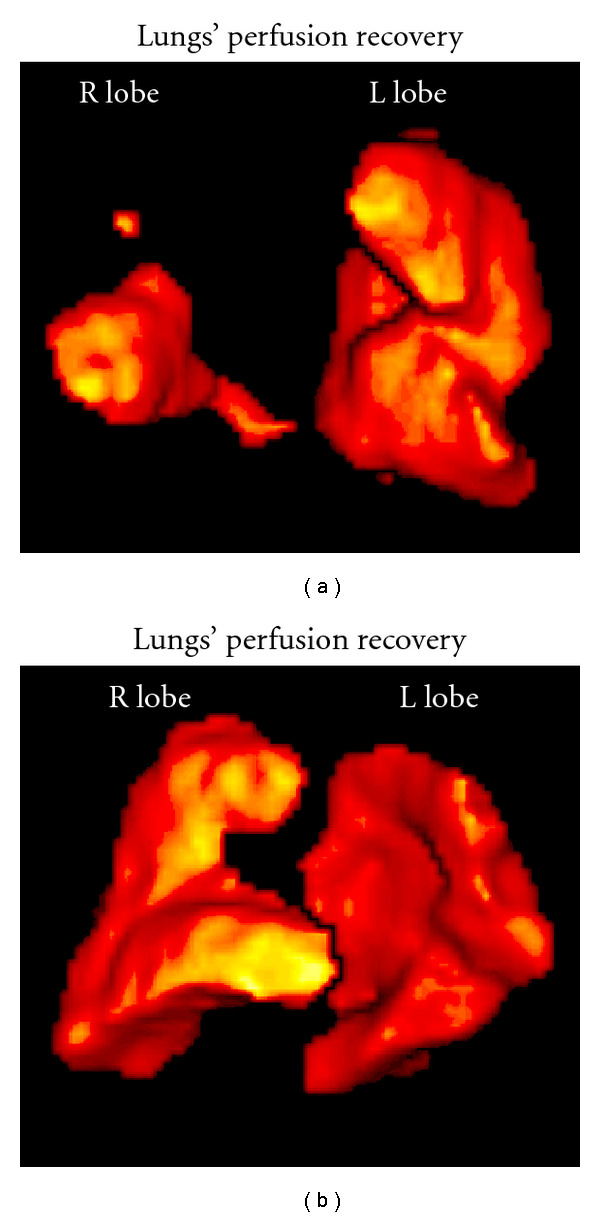
3D SPECT Lungs' FBP reconstruction by Hann filter (critical frequency 0.9) and Chang attenuation correction order 0, coefficient 0.11. (a) 4 hours postevent, Right lobe embolism, R lobe volume 0.66 lt, and total lungs' volume 2.85 lt. (b) 11 days posttherapy, R lobe volume 3.28 lt, and total lungs' volume 5.28 lt. [37] *(modified)*.

A method for lungs' volume determination by SPECT and 3D SPECT images has been demonstrated [37]. Reconstruction was performed by quantitative FBP by Hann filter (critical frequency 0.9) and Chang attenuation correction order 0, coefficient 0.11 in the GE Xeleris2 image processing system. Phantom volume calculations were completed under conditions similar to those of the patients' studies. The method assists to the accurate interpretation of perfusion scans by volume, semi quantitative lung perfusion index [LPI] and pulmonary improvement factor [PIF] determination (Figure 12).

**Thyroid**

SPECT volume estimation is an important tool for dosimetry measurements and radionuclide therapy activity dose determination.

In a comparative study for thyroid volume determination by SPECT, Zaidi (1996) used the third order Butterworth filter with a cut-off frequency equal to 0.4 Nq, for reconstruction of transaxial images of one pixel thickness. Images were obtained without any correction and with two correction methods. A slightly lower value of the attenuation coefficient (*μ* = 0.12 cm^−1^ rather than *μ* = 0.15 cm^−1^ for ^99m^Tc) was accepted better in quantifying thyroid volume by SPECT [38].

Thyroid volume estimations were performed by van Isselt et al., (2003), in patients with Graves' disease [39]. The planar images were subjected to filtering and thresholding, and a standard surface formula was used to calculate the thyroid volume. With SPECT, the iteratively reconstructed thyroid images were filtered, and after applying a threshold method, an automatic segmentation algorithm was used for the volume determinations. Transmission scans, by two gadolinium-153 (^153^Gd) line sources, were reconstructed with FBP and were corrected for down scatter of ^99m^Tc into the ^153^Gd window. For the emission scan an iterative maximum likelihood reconstruction algorithm with attenuation correction and window-based scatter correction as well as resolution recovery was used. For noise reduction, a 3D edge-preserving 3 × 3 × 3-point median filter was applied.

Many times, phantoms of known dimensions have been used in evaluating the accuracy of results in a methodology. Bahk et al., (1998) used an acrylic thyroid phantom in their study for pinhole SPECT imaging in normal and morbid ankles. The phantom was subjected to planar, SPECT and pinhole SPECT acquisitions. The gamma camera system was connected to an Icon data processor that enabled image reconstruction using the FBP algorithm and a Butterworth filter. The *ankles'* SPECT scintigraphy was performed immediately after pinhole scan by 360°detector rotation. The FBP algorithm and a Butterworth filter were used for reconstruction as in the phantom study [40].

## 5. Filter Selection and Standardization

Noise reduction is one of the important tasks in clinical SPECT imaging. One has to be judicious in the selection of filters and its parameters for reducing noise, as there may be some common frequencies in the noise and real image data. Various digital filters (for reducing noise) have been proposed. 

Butterworth, Gaussian, Hamming, Hanning, and Parzen are commonly used SPECT filters during FBP reconstruction, which greatly affect the quality and size accuracy of image. Salihin Yussoff and Zakaria (2009) [7], in a study by a cardiac phantom, had selected 272 combinations of filter parameters and applied on image. Their measurements were used to calculate contrast, signal-to-noise ratio (SNR), and defect size. The different filter types produced myocardial image with different contrast, SNR, and defect size. For contrast and SNR, Gaussian filter was the best, while Parzen filter was the best in producing accurate defect size. However, Butterworth filter was found the best for trade off between contrast, SNR, and defect size accuracy. Selection of filter should consider the qualitative or quantitative type of analysis. For qualitative analysis, high contrast and SNR, Gaussian filter was suggested. The Butterworth filter was suggested for quantitative analysis as it is greatly dependent on both, image quality and size accuracy.

The manufacturers normally supply default filter parameters to the user which may not be relevant in different clinical situations. A phantom study was used in filter parameters standardization and has been compared with those suggested by the vendor. The images were reconstructed using FBP technique with Chang's method (attenuation coefficient = 0.125) attenuation correction. A Ramp filter; sixteen different Hanning filter, thirteen different Metz filter, and nine different Butterworth filter parameters were applied during image reconstruction. Those results did not exactly match with default ones. The filter parameters must be standardized before being put to clinical use is the recommendation [41].

**Figure 13:**
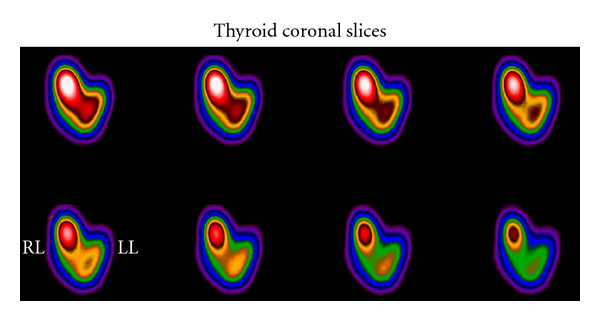
Sequential coronal slices of Thyroid SPECT study. Reconstruction made by FBP and Ramp-Butterworth prefiltering. Right lobe node delineation. *Study is presented courtesy of M. Gavrilelli, MSc, “Medical Imaging Center” Athens, Greece. *

FBP reconstruction has been, for a long time, the only reconstruction algorithm used in SPECT (Figure 13) and is still the reconstruction algorithm recommended for use in National Electrical Manufacturers Association (NEMA) performance tests [42].

The Society of Nuclear Medicine in the Guideline for General Imaging V6.0 9, 2010 gives some recommendations on prefiltering and reconstruction in SPECT imaging [43]. Prefiltering of the projection data in SPECT studies for smoothing in the axial direction must be included. Reconstruction by FBP demands a ramp filter that corrects for the smoothing caused by the back projection process. Filters must be used to restore some of the resolution lost in the reconstruction process. The particular filter that is used depends upon the imaging equipment, the depth of the organ of interest and the radius of rotation. Care should be taken with image enhancement since it is possible to produce artifacts. Though, in iterative reconstruction of SPECT studies the methodology makes it possible to incorporate correction for many physical effects such as nonuniform attenuation correction, scatter reduction or removal, variation of spatial resolution with distance, and so forth, many times the filtering support is necessary. 

## 6. Iterative Reconstruction and Filtering

Iterative image reconstruction methods allow the incorporation of more accurate imaging models rather than the Radon model assumed in the FBP algorithm. These include scatter and attenuation corrections as well as collimator and distance response and more realistic statistical noise models. 

**Figure 14:**
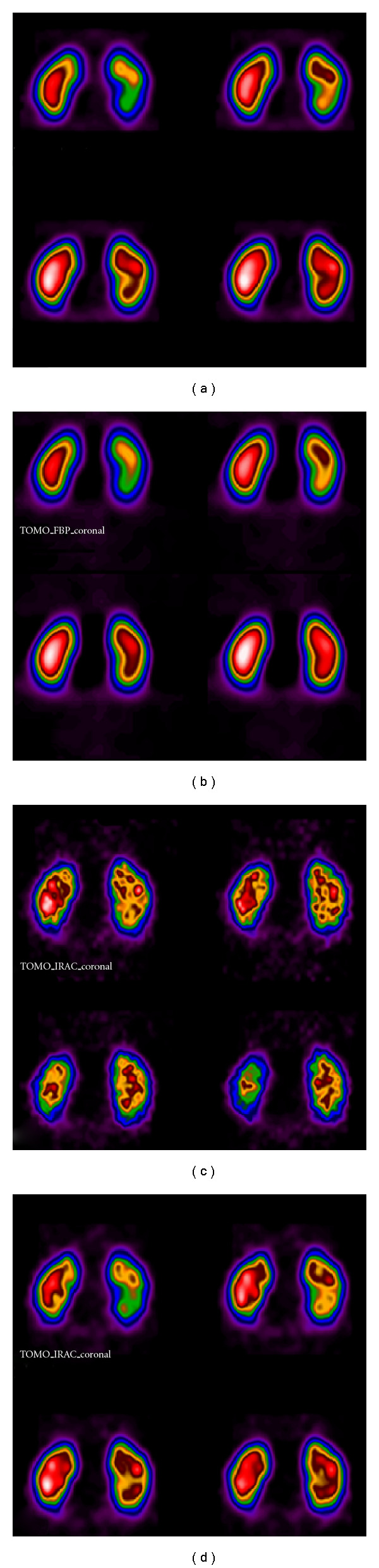
Coronal slices of renal SPECT study. The effect of filtering in smoothing and contrast of SPECT reconstruction, by FBP and Chang attenuation correction (coefficient 0.11) or OSEM iterations is reflected on clinical images. (a) FBP reconstruction, Butterworth filter (critical frequency 0.5 cm^−1^, power 10) (b) FBP reconstruction, Hann filter (critical frequency 0.9 cm^−1^) (c) OSEM 10 subsets/10 iterations (d) OSEM 10 subsets/10 iterations and postfiltering by Butterworth (critical frequency 0.5 cm^−1^, power 10).* Study has been completed in Radiation Physics Unit, Department of Radiology, University of Athens*.

Iterative techniques such as OSEM take into account the Poisson count distribution and the filters are applied mostly postprocessing in 3D. Postfiltering with a Butterworth filter has been shown to result in higher contrasts compared to reconstructions without filtering (Figures 14(c) and 14(d)). 

However, postfiltering with 3D Gaussian filter kernels should be avoided when collimator detector response compensation is included in the reconstruction. Contrast as a function of noise has been studied for prefiltering of ^123^IDAT SPECT images with 2D Gaussian filter kernels and the results showed that contrast as a function of noise is comparable for the prefiltered and nonfiltered OSEM reconstructed images [44]. 

OSEM algorithm convergence properties depend on the activity distribution in the field of view. Resolution properties for OSEM have been studied [45] with different types of regularization. Although different parts of the image converge at different rates, pure and post OSEM filtration achieve reasonably uniform resolution. Inter-iteration filtering (IF OSEM) with smoothing filters, such as a Gaussian, produces images with varying spatial resolution that is dependent on the surrounding activity. It was concluded that the resolution nonuniformity is entirely due to the filtering. A spatially varying filter has been proposed to overcome this problem and to obtain images with nearly uniform resolution. 

Seret in his work [46] in comparison of OSEM and FBP concludes that one might suggest that the number of subsets and iterations chosen should be close to the convergence for all studied regions before quantitative comparisons are made between FBP and OSEM. The number of requested iterations will probably result in images that are too noisy, and a postprocessing filter should be applied.

In a study for comparison of different types of commercial FBP and OSEM SPECT reconstruction software [47] FBP reconstructions were performed by use of the Ramp filter limited at the Nq frequency (0.5 cycle per pixel). Prefiltering of the projections with either the Hanning filter or the order 3 or 6 Butterworth filter was also considered. Three cut-off frequencies (0.20, 0.35, and 0.50 cycles per pixel) were used with the Hanning filter, and 4 cut-off frequencies (0.10, 0.20, 0.35, and 0.50 cycles per pixel) were applied to the Butterworth filter. Most of the types of software were equivalent for FBP or OSEM reconstruction. However, a few differences were observed with some types of software and should be considered when they are used. 

Comparing four sets of coronal images reconstructed by FBP or OSEM and different filters (Figure 14) one evaluates the effect of processing techniques and filtering on the quality of the image.

Using 3D OSEM with suitable AC may improve lesion detectability due to the significant improvement of image contrast. 3D iterative reconstruction algorithms are likely to replace the FBP technique for many SPECT clinical applications. Though, more exact image compensation methods need to be developed and optimal image reconstruction parameters need to be used. The full impact of these methods on quantitative SPECT imaging is yet to be assessed [48].

An efficient postprocessing method to compensate for scattering and blurring effects in inhomogeneous media was presented by Yan and Zeng (2008) [49]. The major challenge of the method is to accurately estimate the 2D point spread function (PSF) in the image domain. From the clinical aspect, the implementation of the method is faster than the iterative reconstruction-based compensation method. This method is developed in two dimensions and does not consider scattered photons from out-of-plane sources. Future work will possibly include modelling the scattering with a 3D-PSF and a comparison between 3D-PSF method and 3D-OSEM could be done. 

## 7. Discussion and Conclusion

SPECT has become an important diagnostic tool in nuclear medicine. SPECT images show characteristic anatomical and functional information of the structures and the tissues. The quality of the image depends on several factors such as spatial resolution (detail or sharpness), contrast and noise (statistical and structure). 

One of the most important factors that greatly affect the quality of clinical SPECT images is image filtering. Image filtering is a smoothness process for noise removal and resolution recovery. A number of filters have been designed and are available in the reconstruction of tomographic images. All of them are characterized by two parameters: the cut-off frequency and the order. The same filter with different parameters can affect variously the image quality. The type of the filter and the application of the filter parameters cannot be generalized in all types of clinical SPECT studies. 

The selection of the optimal filter and the determination of filter parameters for any individual case remains one of the main problems of filtering in SPECT image processing. Especially the selection of the cut-off frequency is very important in order to reduce noise and preserve the image details. Proper filter selection is significant for the improvement of the image quality and therefore for the diagnostic evaluation. However, no filter is perfect and there is no specific filter for all applications. In the literature there are limited reports for the choice of the appropriate filter parameters in a certain SPECT examination, as the findings of filter application per organ reconstruction cannot be generalized.

Nowadays, FBP reconstruction is progressively replaced with the OSEM-iterative reconstruction algorithm. Unlike FBP, OSEM is not a linear algorithm, and the reconstructed contrast depends on the true contrast and on object size. Moreover, FBP is still faster than OSEM and remains widely used in clinical practice [40]. Iterative reconstruction methods have seen a significant growth in tomographic reconstruction because of the increased computerizing speed. Iterative reconstruction algorithms produce accurate images of radioactive distribution and seem to be more sensitive than FBP technique [47]. Further development in iterative reconstruction methods will be very promising in improving image quality. Alzimami et al. (2009) have demonstrated an improved performance of the new 3-D OSEM method compared to FBP, particularly for low count statistics. It is necessary, though, optimal image reconstruction parameters to be used for the comparison of the full potential of these methods and evaluation of their impact on quantitative SPECT imaging [50]. 

 FBP and OSEM are generally both available on all SPECT processing software developed by gamma camera manufacturers and the nuclear medicine processing software companies. The SPECT filters can greatly affect the quality of clinical images by their degree of smoothing. Proper filter selection and adequate smoothing helps the physician in results' interpretation and accurate diagnosis. 

## Figures and Tables

**Figure 2:**
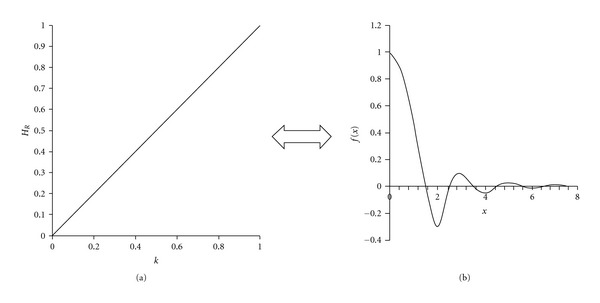
The Ramp filter: (a) Ramp filter in frequency domain. (b) Ramp filter in spatial domain [3].
